# Integrated Transcriptomic and Biochemical Analyses Reveal the Root Development-Promoting Mechanism of *Piriformospora indica* on Blueberry Under Tap Water Irrigation

**DOI:** 10.3390/plants14233646

**Published:** 2025-11-29

**Authors:** Sijian Guo, Pengyan Qu, Shitao Du, Rui Liu, Yongyan Zhang, Chunzhen Cheng

**Affiliations:** 1 College of Horticulture, Shanxi Agricultural University, Taigu, Jinzhong 030801, China; 2 Shanxi Key Laboratory of Germplasm Improvement and Utilization in Pomology, Taiyuan 030031, China

**Keywords:** *Piriformospora indica*, blueberry, mild salt stress, transcriptomic analysis, molecular mechanism

## Abstract

*Piriformospora indica*, a broad-spectrum plant growth-promoting fungus, has been successfully applied in blueberry (*Vaccinium corymbosum* L.). In this study, through an integrated transcriptomic and biochemical analyses, we investigated the effects of *P. indica* colonization on blueberry root growth under long-term tap water (EC ≈ 1500 μs/cm) irrigation. Comparative transcriptomic analysis revealed that *P. indica* colonization greatly influenced the expression of genes involved in RNA biosynthesis, solute transport, response to external stimuli, phytohormone action, carbohydrate metabolism, cell wall organization, and secondary metabolism pathways. Consistently, the fungal colonization significantly improved the nutrient absorption ability, and increased the contents of sucrose, starch, trehalose, total phenolic, total flavonoids, and indole-3-acetic acid (IAA), while suppressing the accumulations of jasmonic acid (JA), abscisic acid (ABA), 1-aminocyclopropane-1-carboxylic acid (ACC), and strigolactone (SL) in blueberry roots. Quantitative real-time PCR verification also confirmed the fungal influences on genes associated with these pathways/parameters, such as auxin homoeostasis-associated *WAT1*, cell wall metabolism-related *EXP*, phenylpropanoid biosynthesis-related *PAL* and *CHS*, carotenoid degradation-related *CCD8*, transportation-related *CNGC*, trehalose metabolism-related *TPP,* and so on. Our study demonstrated that *P. indica* improved blueberry adaptability to mild salt stress by synergistically regulating cell wall metabolism, secondary metabolism, stress responses, hormone homeostasis, sugar and mineral element transportation, and so on.

## 1. Introduction

*Piriformospora indica* (also named *Serendipita indica*), an arbuscular mycorrhizal fungus (AMF)-like fungus [1], is capable of colonizing not only the host plants of AMFs but also Brassicaceae family plants, which AMFs cannot colonize [2]. *P*. *indica* can be axenically cultured on a many synthetic and semi-synthetic media [3], making its application much easier than AMF. The growth-promoting effects of *P. indica* have been confirmed in many of its host plants [4], including both herbaceous plants [5,6,7] and woody plants [8,9,10]. *P. indica* can also enhance the tolerance of host plants to various stresses, including salinity. Evidence reveals that *P. indica* colonization can enhance the salt tolerance of host plants by enhancing antioxidant enzyme activity [11], reducing oxidative damage [12,13,14], adjusting ion homeostasis and transporter-related genes expression [15,16], and improving nutrient uptake [13].

Blueberry (*Vaccinium corymbosum* L.) has a shallow and hairless root system [10]. Its water and nutrient absorption ability is weak, particularly under stressful environments. To compensate for this disadvantage, blueberry roots usually form symbiotic relationships with mycorrhizal fungi [10]. Recently, several endophytic fungi, such as *Penicillium chrysogenum*, *P. brevicompactum* [17], and dark septate endophytes [18,19], have been applied in blueberry, exhibiting significant plant growth-promoting and/or stress tolerance-enhancing effects. It is worth noting that the colonization of *P. indica* in blueberry roots demonstrated multifaceted benefits. The fungal colonization significantly improved maximum shoot length and total shoot biomass [20], greatly promoted the rooting of cuttings and the growth of cutting seedlings [10], and significantly enhanced drought tolerance [21] and *Phytophthora cinnamomi* resistance in blueberry plants [20].

Blueberry plants are highly sensitive to soil salinity [22]. Under salt stress, the growth and productivity of blueberry plants are greatly reduced [23]. Recently, many families have grown blueberry plants on patios or balconies and in yards, where tap water is often used for irrigation. However, prolonged irrigation with tap water induces a significant rise in soil electrical conductivity (EC), leading to impaired blueberry plant growth. Given the demonstrated role of *P. indica* in promoting plant growth under salt stress [11,12,13,14,15,16], this study investigated the effects of *P. indica* colonization on the root growth of ‘Legacy’ blueberry plants under tap water (EC ≈ 1500 μs/cm) irrigation (18 months). To explore the underlying mechanisms, we conducted comparative transcriptomic analysis of *P. indica*-colonized (PI) and non-colonized control (CK) blueberry roots. Furthermore, we validated the expression of 15 differentially expressed genes (DEGs) involved in significantly enriched pathways—such as phytohormone signaling, solute transport, cell wall metabolism, stress response, and secondary metabolism—using quantitative real-time PCR (qRT-PCR). We also determined biochemical parameters closely linked to these pathways, including profiling of carotenoid and the contents of sucrose, starch, trehalose, total phenolics, flavonoids, phytohormones, mineral elements, and so on. This study will be helpful in understanding how *P. indica* promotes blueberry root development under long-term tap water irrigation conditions.

## 2. Results

### 2.1. P. indica Colonization Improved the Root Biomass of Blueberry Plants Under Long-Term Tap Water Irrigation

Three months post *P. indica* inoculation, PCR analysis was conducted to examine its colonization in blueberry roots. *Pitef1* fragments were successfully amplified from roots of all the *P. indica*-inoculated blueberry plants (Figure S1), demonstrating effective colonization by the fungus. The root fresh weight of the PI group was significantly higher than that of the CK group (Table 1), accounting for 1.39-fold (*p* < 0.05). Although no significant root dry weight difference was identified between CK and PI, the average root dry weight of PI seedlings was approximately 1.24-fold that of CK plants (*p* > 0.05). Moreover, the root activity of PI accounted for about 1.41-fold that of CK (*p* > 0.05).

EC measurements showed that substrate EC reached ≈600, 1200, and 1500 μS/cm after 6, 12, and 18 months of tap water irrigation, respectively. Because EC > 1500 μS/cm is known to impair blueberry root development [24], the 18-month treatment imposed mild salt stress on the blueberry plants. Thus, *P. indica* colonization promoted root growth under this tap water-induced mild salt stress.

### 2.2. GO and KEGG Enrichment Analysis of DEGs

Through RNA-seq, we obtained about 42.81 Gb of high-quality clean data from six blueberry root cDNA libraries (three replicates for both CK and PI groups), with each sample yielding over 6.25 Gb high-quality data and genome mapping ratio ranging from 83.89% to 89.54% against the *V*. *corymbosum* cv. Draper v1.0 reference genome (Supplementary Table S1). In total, 101,410 genes, including 95,018 known genes and 6392 novel genes, were identified to be expressed in blueberry root. Among them, 3315 genes were identified as differentially expressed genes (DEGs) in PI compared to CK, including 2753 down-regulated and 562 up-regulated genes.

**Figure 1:**
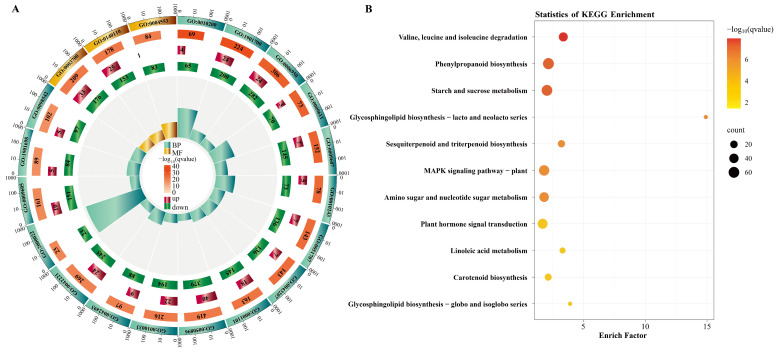
GO and KEGG enrichment analysis of DEGs identified between CK and PI blueberry roots. (**A**) GO enrichment horizontal bar chart for the top 20 enriched terms. Bar colors indicate GO categories: orange for molecular function (MF) and blue for biological process (BP). The numbers in parentheses represent the count of differentially expressed genes (DEGs) annotated to each term. The fill colors of the bars (red or green) reflect the predominant regulation direction of these DEGs: red indicates up-regulation, while green indicates down-regulation. (**B**) KEGG pathways significantly enriched among DEGs. Larger dots indicate more DEGs.

Gene ontology (GO) enrichment analysis of DEGs showed that they were significantly enriched in 406 biological processes (BPs) terms, 25 cellular components (CCs) terms, and 166 molecular functions (MFs) terms (*q* < 0.05, Benjamini–Hochberg (BH) method) (Supplementary Table S2). Of the top 20 enriched GO terms, several were related to stress responses, including response to stress (GO:0006950, 306 DEGs), response to stimulus (GO:0050896, 419 DEGs), and response to external stimulus (GO:0009605, 161 DEGs) (Figure 1A). This finding suggested that the salt stress responses caused by tap water irrigation in blueberry roots were altered by *P. indica* colonization.

Kyoto Encyclopedia of Genes and Genomes (KEGGs) enrichment analysis revealed that the DEGs between CK and PI were significantly enriched in 11 pathways (*q* < 0.05, BH method), including phenylpropanoid biosynthesis (ko00940, 65 DEGs), starch and sucrose metabolism (ko00500, 61 DEGs), MAPK signaling pathway–plant (ko04016, 54 DEGs), plant hormone signal transduction (ko04075, 50 DEGs), amino sugar and nucleotide sugar metabolism (ko00520, 44 DEGs), valine/leucine/isoleucine degradation (ko00280, 37 DEGs), sesquiterpenoid and triterpenoid biosynthesis (ko00909, 18 DEGs), carotenoid biosynthesis (ko00906, 16 DEGs), and so on (Figure 1B). These findings indicated that *P. indica* colonization strongly influenced primary and secondary metabolic and signaling pathways in blueberry roots.

### 2.3. MapMan Annotation and PageMan Enrichment Analysis of DEGs

Functional annotation of DEGs was further conducted using MapMan v3.6.0. Among the 3315 DEGs, 1443 (43.5%) were classified as ‘unannotated’, while the remaining annotated genes were systematically categorized into 31 level-1 BINs. It is worth noting that most of the annotated DEGs were enriched in enzyme classification (624 DEGs), RNA biosynthesis (365 DEGs), protein modification (200 DEGs), solute transport (193 DEGs), protein homeostasis (160 DEGs), phytohormone action (109 DEGs), and cell wall organization (98 DEGs) BIN pathways (Supplementary Table S3). PageMan (version 3.5.0.) enrichment analysis results showed that these DEGs were significantly enriched in 35 pathways from 11 level-1 BINs, including RNA biosynthesis, solute transport, response to external stimuli, phytohormone actions, carbohydrate metabolism, cell wall organization, multi-process regulation, coenzyme metabolism, protein modification, protein homeostasis, and secondary metabolism (Table 2).

Most RNA biosynthesis-related DEGs were transcription factor (TF) genes. Among them, *AP2/ERFs* (73 DEGs: 6 up-regulated, 67 down-regulated), *DREBs* (43 DEGs: 3 up-regulated, 40 down-regulated), and *WRKYs* (41 DEGs: all down-regulated) were the largest top three groups. Additionally, 21 *bHLHs*, 16 *C2H2s*, 15 *TIFYs* (all down-regulated), 9 *DOFs* (all up-regulated), and 6 *PLATZs* (all up-regulated) were annotated as RNA biosynthesis-related DEGs (Supplementary Table S4).

Solute transport-related DEGs included 20 *P2B-type Ca^2+^-ATPase* genes, 14 *phosphate transporter* genes, 10 *nitrate transporter* genes, 7 *Ca^2+^ exchanger* genes, 5 *K^+^ transporter* genes, 4 *AMT family* members, 3 *MATE family* members, 43 *ZIP family* genes, and so on (Supplementary Table S5). Notably, 43 of the 44 primary active transport-related DEGs and all nine *nucleotide sugar transporter* (UUAT) genes were down-regulated. In contrast, seven *walls are thin 1* (*WAT1*) gene, two *hexose carrier* genes (*HEX6*), two *MIP* (*major intrinsic protein*) *family transporters*, and three *cyclic nucleotide-gated channel* (*CNGC*) genes were up-regulated in PI roots. These results suggested that *P. indica* colonization significantly alters solute transport and nutrient acquisition in blueberry roots.

A total of 91 DEGs (15 up-regulated, 76 down-regulated) were enriched in the external stimuli response category (Supplementary Table S6). Notably, three DEGs encoding the key signaling protein NSP2 (nodulation signaling pathway 2) in the rhizobial symbiosis signaling pathway were up-regulated in PI.

Of the 109 phytohormone action-related DEGs (Supplementary Table S7), 27 were up-regulated and 82 were down-regulated. All ethylene biosynthesis and jasmonic acid (JA) signaling-related DEGs were down-regulated. Five JA conjugation- and degradation-related *CYP94Cs* genes were down-regulated. However, eight strigolactone-related DEGs, three ABA-related *carotenoid cleavage oxygenase 8* (*CCD8*) genes, and two salicylic acid (SA)-related *PR-1* genes were up-regulated in PI.

There were 51 DEGs (4 up-regulated, 47 down-regulated) enriched in the carbohydrate metabolism pathway (Supplementary Table S8). Of them, seven DEGs encoding trehalose-6-phosphate phosphatase (TPP) were significantly down-regulated in PI.

A total of 98 DEGs (18 up-regulated and 80 down-regulated) were enriched in cell wall organization (Supplementary Table S9). Notably, ten DEGs encoding alpha-class expansin (EXPA) family proteins were all up-regulated in PI.

Among the 48 secondary metabolite biosynthesis-related DEGs, all the 26 terpenoid backbone biosynthesis- and metabolic-related DEGs were down-regulated in PI. However, four DEGs encoding phenylalanine ammonia-lyase (PAL), two DEGs encoding chalcone synthase (CHS), and two DEGs encoding 2-hydroxyisoflavone dehydratase (HID) were significantly up-regulated (Supplementary Table S10).

The circadian clock system pathway was significantly enriched by nine DEGs, including seven up-regulated genes encoding time-of-day-dependent transcriptional repressors (PRRs). Of the protein modification-related DEGs, there were 18 genes encoding LRR-XII receptors (15 up-regulated and 3 down-regulated).

### 2.4. Gene Expression Validation Results of Selected DEGs

**Figure 2:**
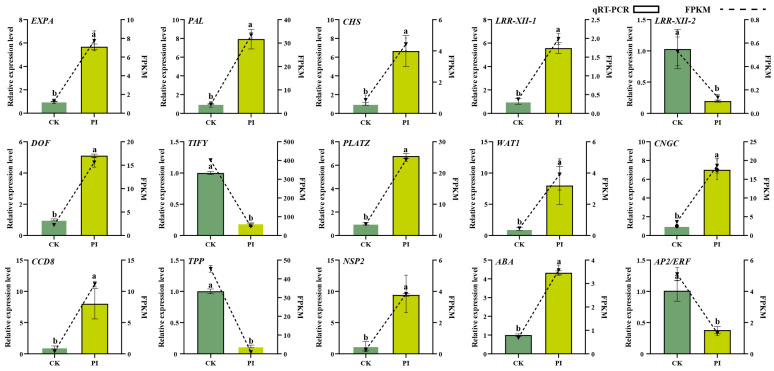
Gene expression validation of 15 selected DEGs. CK: non-colonized control roots; PI: *P. indica*-colonized blueberry roots. Error bars represent the standard error (SE) of three biological replicates. Different letters (a, b) above the columns indicate significant differences between non-colonized (CK) and *P. indica*-colonized (PI) groups (Student’s *t*-test, *p* < 0.05).

Quantitative real-time PCR (qRT-PCR) was used to validate the expression of fifteen DEGs, including *EXPA*, *PAL*, *CHS*, *leucine-rich repeat receptor kinases* (*LRR-XII-1* and *LRR-XII-2*), *DNA-binding with one finger transcription factor* (*DOF*), *TIFY transcription factor family* (*TIFY*), *PLATZ transcription factor* (*PLATZ*), *WAT1*, *GNGC*, *CCD8*, trehalose-phosphate phosphatase (TPP), *NSP2*, *abscisic acid biosynthesis enzyme* (*ABA*), and *APETALA2/ethylene-responsive factor* (*AP2/ERF*) genes. Results showed that their expression patterns were consistent with the RNA-seq data (Figure 2), indicating the reliability of our transcriptomic data.

### 2.5. Validation of Key Biochemical Parameters Associated with Pathways Significantly Enriched by DEGs

**Figure 3:**
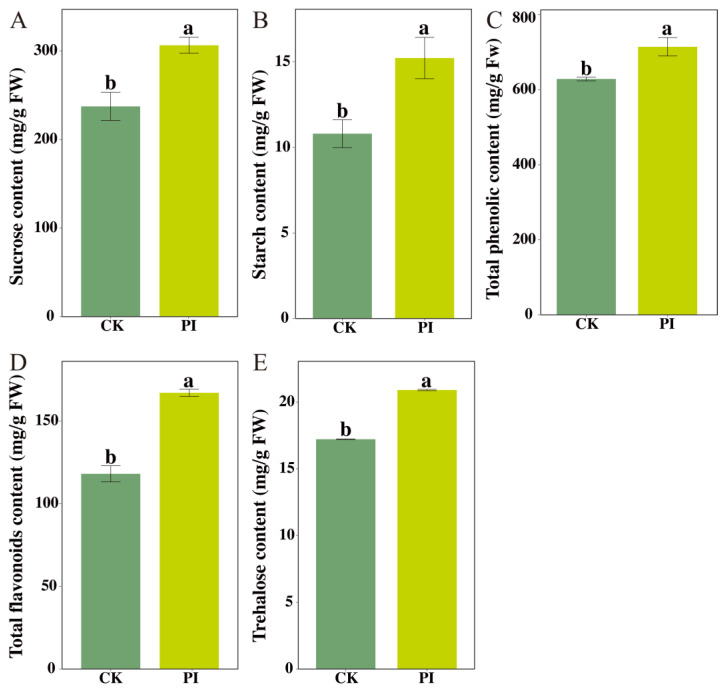
Influences of *P. indica* colonization on the accumulations of sucrose (**A**), starch (**B**), total phenolic (**C**), total flavonoids (**D**), and trehalose (**E**) in blueberry roots. FW: fresh weight. Different letters (a, b) above the columns indicate significant differences between non-colonized (CK) and *P. indica*-colonized (PI) groups (Student’s *t*-test, *p* < 0.05).

KEGG enrichment analysis revealed that the phenylpropanoids biosynthesis pathway (ko00940) and starch and sucrose metabolism pathway (ko00500) were significantly enriched by DEGs. Consistently, PageMan enrichment analysis demonstrated that DEGs were significantly enriched in secondary metabolism (including terpenoid metabolism) and carbohydrate metabolism (particularly trehalose metabolism). To validate these findings, the contents of sucrose, starch, total phenolics, total flavonoids, and trehalose in blueberry roots were measured (Figure 3). *P. indica* colonization significantly increased sucrose, starch, and total phenolics contents by 29.07%, 40.88%, and 13.63%, respectively. Additionally, the total flavonoids and trehalose contents in PI were also significantly higher than those in CK, being 1.42- and 1.21-fold of it, respectively.

KEGG enrichment analysis revealed that the carotenoid biosynthesis pathway was significantly enriched by DEGs. Our carotenoid profiling analysis identified a total of 34 carotenoid compounds in blueberry roots, including 5 carotenes and 29 xanthophylls. The total carotene content in PI was approximately 1.23-fold of CK, while its total xanthophylls content accounted for about 97.07% of CK. Seven carotenoid compounds were identified as differentially accumulated metabolites (DAMs) between CK and PI. Compared to CK, the contents of lycopene, (E/Z)-phytoene, and zeaxanthin in PI significantly increased, while the contents of palmitic acid amaranthin, palmitic acid lutein, zeaxanthin–oleate–palmitate, and palmitic acid *β*-cryptoxanthin significantly decreased (Supplementary Table S11). Notably, all four down-regulated DAMs belong to xanthophylls.

**Figure 4:**
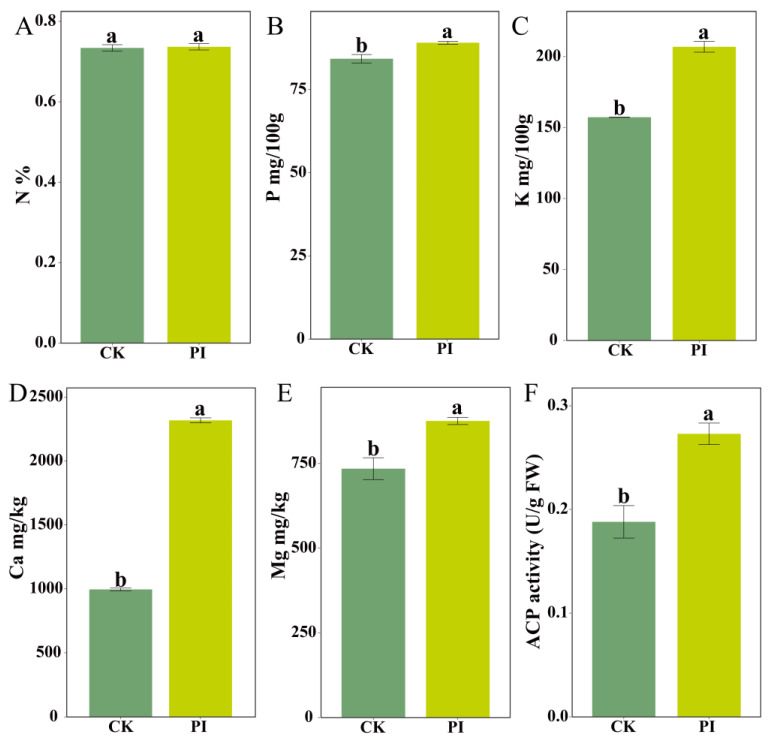
Mineral element contents (**A**–**E**) and ACP activity (**F**) in roots of *P. indica*-colonized (PI) and non-colonized (CK) blueberry plants. (**A**–**E**) Contents for nitrogen (N), phosphorus (P), potassium (K), calcium (Ca), and magnesium (Mg), respectively. Different lowercase letters (a, b) above the bars indicate statistically significant differences between CK and PI groups (Student’s *t*-test, *p* < 0.05).

PageMan enrichment analysis showed that the solute transport pathway was significantly enriched by numerous DEGs encoding mineral-element-related transporters. To investigate the effects of *P. indica* colonization on mineral element homeostasis, we quantified nitrogen (N), phosphorus (P), potassium (K), calcium (Ca), magnesium (Mg) contents, and acid phosphatase (ACP) activity in blueberry roots. Compared to CK, PI roots exhibited significant increases in K, Ca, P, and Mg contents (*p* < 0.05), representing approximately 1.32-, 1.13-, 1.06-, 1.19-fold increases, respectively. Moreover, the root ACP activity in PI was 1.46-fold of CK (*p* < 0.05). These results indicated that *P. indica* colonization markedly enhanced mineral element acquisition efficiency in blueberry roots (Figure 4).

### 2.6. Determination of Endogenous Phytohormone Contents in CK and PI Blueberry Roots

**Figure 5:**
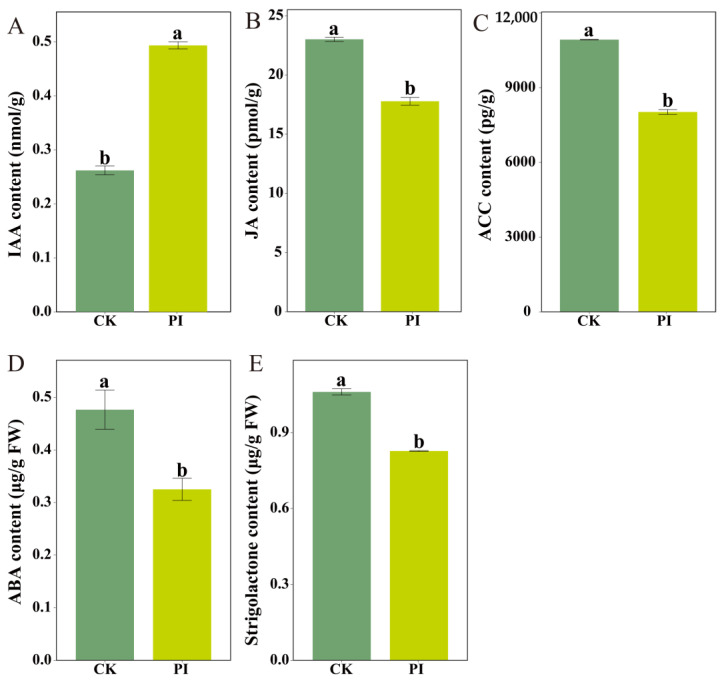
Influences of *P. indica* colonization on the accumulations of phytohormones in blueberry roots. (**A**–**E**) Contents of indole-3-acetic acid (IAA), jasmonic acid, 1-aminocyclopropane-1-carboxylic acid content (ACC), abscisic acid content (ABA), and strigolactones (SLs), respectively. FW: fresh weight. Different letters (a, b) indicate significant differences between non-colonized (CK) and *P. indica*-colonized (PI) groups (Student’s *t*-test, *p* < 0.05).

GO enrichment analysis showed that “jasmonic acid-mediated signaling pathway” term was significantly enriched by DEGs. KEGG enrichment results revealed significant enrichment in the ‘hormone signal transduction’ pathway. PageMan analysis also revealed significant enrichment of JA- and strigolactones (SLs)-related pathways. These findings indicated that *P. indica* colonization greatly altered phytohormone metabolism in blueberry roots. To verify this, we determined the contents of indole-3-acetic acid (IAA), JA, 1-aminocyclopropane-1-carboxylic acid (ACC), abscisic acid (ABA), and SL in CK and PI roots. The IAA content in PI roots was significantly higher than that in CK (*p* < 0.05), accounting for approximately 1.88-fold of it. Meanwhile, the JA, ACC, ABA, and SL contents were significantly reduced (*p* < 0.05) in PI roots, accounting for 77.26%, 73.43%, 68.18%, and 54.73% of CK (Figure 5), respectively.

## 3. Discussion

Long-term tap water irrigation can increase soil EC, thereby negatively affecting root system development and plant growth [24]. Our study demonstrated that *P. indica* colonization significantly improved the root fresh weight and increased the root activity and root dry weight of blueberry plants. These results indicated that fungal colonization promoted blueberry root growth under mild salt stress condition. To further explore the root growth-promoting mechanism of *P. indica* in blueberry under long-term tap water irrigation, integrated transcriptomic and biochemical analysis was performed. Results revealed that fungal colonization greatly influenced several biological processes in blueberry roots, including symbiotic signaling, RNA biosynthesis, cell wall metabolism, metabolite and mineral element accumulation, stress responses, and sugar transport metabolism.

### 3.1. P. indica Colonization Modulates the Symbiotic Signaling Pathway and Cell Wall Metabolism in Blueberry Roots

NSP1 and NSP2, members of the GRAS transcription factor family [25], play pivotal roles in symbiotic signaling pathways [26]. *NSP2* and *bHLH476* function as direct targets of cytokinin signaling and play crucial roles in symbiotic nodulation [27]. Amino acid polymorphisms within the conserved VHIID motif of NSP2 significantly modulate symbiotic signaling and nodule morphogenesis [28]. In *Medicago truncatula* and *Oryza sativa*, the *nsp1/nsp2* double mutants exhibited significantly reduced colonization rates of AMF [29]. In this study, the significant up-regulation of *NSP1* and *NSP2* in PI blueberry roots suggested that these genes may act as key regulators at the symbiotic interface, potentially facilitating fungal accommodation through modulation of plant cell wall remodeling or nutrient exchange pathways.

Accumulating evidence indicates that *α-expansin* plays a pivotal role in plant developmental processes [30], mediating cell wall extension [31,32,33], thereby promoting cell expansion [34,35]. In *Arabidopsis*, *AtEXPA7* and *AtEXPA18* are specifically expressed in root hairs, and their overexpression promotes root hair initiation and enhances root growth [36]. *AtEXPA17* overexpression enhanced lateral root formation, whereas its knockdown reduced this developmental process [37]. The overexpression of *OsEXPA7* in rice not only modulated the expression of *OsJAZs* in the JA pathway and *BZR1/GE* in the brassinosteroid signaling pathway [38], but also significantly reduced sodium (Na^+^) and potassium (K^+^) accumulation in both leaves and roots [39]. *OsEXPA17* is specifically expressed in rice root hairs, and its mutation significantly impairs root hair elongation [40]. In our study, the significantly higher expression levels of ten *α-expansin* genes in PI roots suggested that *P. indica* promotes root growth and improves root architecture through enhancing the *α-expansin*-mediated cell wall extension.

### 3.2. P. indica Colonization Promotes Blueberry Root Development Through Modulating Carotenoid and Phytohormone Metabolism, Improving Secondary Metabolism, and Strengthening Stress Responses

Phytohormones play pivotal roles in regulating root development and plant growth [41,42]. In this study, *P. indica* colonization significantly increased the IAA content and up-regulated the expression of seven *WAT1* genes in blueberry roots. *WAT1* is a member of the MtN21 family [43,44], which is primarily associated with amino acid and auxin transport [45]. These results suggested that *P. indica* colonization may activate auxin transport through up-regulating *WAT1* expression, thereby promoting IAA accumulation and ultimately improving the development of blueberry roots.

*P. indica* colonization significantly decreased the JA and ACC contents in blueberry roots, which was consistent with our previous findings in blueberry cuttings [10]. Moreover, our study found that all ethylene biosynthesis- and JA signaling-related DEGs were down-regulated in PI roots. These suggested that fungal colonization promotes root development by suppressing the biosynthesis of JA and ethylene [11].

Carotenoid-derived metabolites play a pivotal role in mediating signaling crosstalk and facilitating symbiotic association establishment with AMF [46]. The present study revealed that carotenoid degradation-related *CCD8* genes were significantly up-regulated in PI roots, suggesting that *P. indica* colonization exacerbated carotenoid catabolism. Carotenoids serve as precursors for the phytohormones SLs and ABA, both of which are key regulators of root growth and development [47,48]. Our study showed that the SL and ABA contents in PI blueberry roots significantly decreases. In rice, ethylene induces the expression of *MHZ5* and the biosynthesis of neoxanthin (a precursor of ABA), which collectively suppress root growth in seedlings [49]. SLs, a class of sesquiterpenoid lactones, act as root-derived chemical signals that modulate both symbiotic and parasitic interactions between AMF and their host plants [50]. SLs induce AM fungal spore germination and hyphal branching [51], while positively modulating root hair elongation/density and suppressing lateral root formation. Collectively, these results demonstrate that *P. indica* colonization profoundly altered carotenoid metabolism and phytohormone metabolism in blueberry roots.

Our study also found that *P. indica* colonization significantly increased the contents of total phenolics and flavonoids and greatly influenced the expression of phenylpropanoid biosynthesis/secondary metabolism-related genes. These findings indicated that fungal colonization improved the growth of blueberry under tap water irrigation by mediating the biosynthesis of secondary metabolites. Additionally, enrichment analysis of DEGs revealed that genes involved in several stress response terms and responses to external stimulus pathways were significantly enriched, indicating that *P. indica* colonization influenced stress responses to mild salt damage caused by long-term tap water irrigation.

### 3.3. P. indica Promotes Blueberry Root Development by Enhancing Mineral Element Absorption and Sugar Transport Metabolism

*P. indica* enhances the uptake of mineral nutrients and other essential substances in host plants under both optimal and stressful conditions. In this study, *P. indica* colonization significantly increased concentrations of K, Ca, P, and Mg in blueberry roots, accompanied by substantial modulation of genes associated with solute transporter pathways. CNGCs play pivotal roles in diverse physiological processes in plants [52] and are recognized as critical calcium-permeable channels [53,54,55]. The *MtCNGC15a/b/c* genes modulate nuclear calcium release and play critical roles in rhizobial and mycorrhizal symbiotic processes in *M. truncatula* [56]. The CNGC family may regulate root hair tip growth, with *CNGC5*, *CNGC6*, *CNGC9*, and *CNGC14* functioning as potential modulators of this process [57,58,59]. Mutations in *CNGC5*, *CNGC6*, and *CNGC9* resulted in shorter root hairs [58], whereas defects in *CNGC14* led to impaired root hair growth [59,60]. Our study revealed that *P. indica* colonization significantly up-regulated the expression of multiple *CNGC* genes and increased calcium content in blueberry roots, suggesting that *P. indica* promotes nutrient uptake through induction of *CNGC* gene expression, thereby facilitating root system development.

The symbiotic interaction with *P. indica* enhanced the phosphorus uptake of oilseed rape by increasing phosphatase activity and up-regulating the expression of the phosphate transporter gene *BnPht1;4* [61]. This improvement was accompanied by significantly elevated accumulation of multiple essential elements [62]. *P. indica* colonization significantly enhanced wheat plant biomass and elevated zinc (Zn) and iron (Fe) concentrations in both stems and roots, with these micronutrients preferentially accumulating of in roots rather than in stems [47], thereby contributing to improved grain yield. This study demonstrated that *P. indica* colonization significantly increased both ACP activity and P content in blueberry roots, indicating that fungal colonization promoted the phosphorus absorption ability of blueberry plants.

The hexose transporter HEX6 belongs to the sugar transporter family, which plays a critical role in carbohydrate allocation and stress responses in plants [63]. In this study, *P. indica* significantly up-regulated the expression of two *HEX6* genes, as well as increased sucrose and starch accumulation in blueberry roots. These findings suggest that *P. indica* colonization may enhance hexose transporter activity through induction of *HEX6* expression, thereby promoting carbohydrate storage. Trehalose produced by *P. indica* activates ABA signaling, forming a positive feedback loop that enhances drought resilience and yield stability in wheat [64]. Notably, the trehalose content in *P. indica*-colonized blueberry roots was markedly elevated. However, the trehalose metabolism pathway was significantly enriched by seven down-regulated *TPP* genes. This paradoxical pattern may reflect feedback inhibition triggered by excessive trehalose accumulation.

## 4. Materials and Methods

### 4.1. Plant Materials and P. indica Inoculation

Healthy and uniform ‘Legacy’ blueberry plants (with plant height of 50–60 cm) grown in pots (Diameter = 28 cm; Height = 28 cm) filled with growth substrates (EC ≈ 210 μS/cm; pH = 5.0; Pindstrup, Ryomgård, Denmark) were divided into PI group and CK group. Each group consisted of at least 30 blueberry plants. Blueberry plants of the PI group were watered with 200 mL *P. indica* fermentation solution three times according to Cheng et al. [65], while CK group plants were watered with an equal volume of potato dextrose broth (PDB) as controls. The *P. indica* strain (DSM11827) used for blueberry inoculation was provided by professor KaiWun-Yeh of Taiwan University and maintained in our laboratory. After inoculation, blueberry plants were grown in a controlled growth chamber with a temperature of 25 ± 2 °C, a relative humidity range of 60–80%, a photoperiod of 16 h light/8 h dark, and a light intensity of 1500 lx. Blueberry plants were irrigated with tap water every three days (pH 8.0, [EC] ≈ 450 μS/cm). To maintain low soil pH, 0.3~0.6 g/kg of finely powdered sulfur (particle size < 0.15 mm) was added to substrates quarterly [66]. By using the electrode method, the EC of substrates was monitored at 6, 12, and 18 months post tap water irrigation.

Three months post *P. indica* inoculation, genomic DNA was extracted from blueberry roots using a Plant Genomic DNA Extraction Kit (TIANGEN Biotech Co., Ltd., Beijing, China) and used as the template for PCR amplification of the *Pitef1* gene [67].

### 4.2. Determination of Root Fresh and Dry Weight and Root Activity

At 18 months post *P. indica* inoculation, root fresh weight and root dry weight of blueberry plants from CK and PI groups were measured. Root fresh weight of blueberry plants was first measured using an electronic balance (HAT-A+100, Huazhi (Fujian) Electronics Technology Co., Ltd., Putian, China). Then, root samples were oven-dried at 65 °C to constant weight and weighted. Moreover, using the 2,3,5-Triphenyltetrazolium Chloride (TTC) reduction assay, root activities of CK and PI blueberry plants were measured [68]. For each parameter, six blueberry plants from each group were used.

### 4.3. Transcriptome Sequencing

Total RNA was extracted from roots of CK and PI groups using the Trizol method. After assessing RNA purity, concentration, and integrity using NanoDrop 2000 spectrophotometer (Wilmington, DE, USA) and Agilent 2100 Bioanalyzer/LabChip GX system (Santa Clara, CA, USA), high-quality RNA samples were subjected to RNA-Seq analysis using the Illumina NovaSeq6000 sequencing platform at Beijing Biomarker Biotechnology Co., Ltd. (Beijing, China), with three biological replicates per group. Clean reads were aligned to the *Vaccinium corymbosum* L. genome (cv. Draper v1.0, https://www.vaccinium.org/analysis/49 (accessed on 25 July 2023)) with HISAT2. Mapped reads were then assembled using StringTie v3.0.0 and compared against the existing annotation to identify novel genes. Transcripts encoding peptides shorter than 50 amino acids or containing only a single exon were discarded.

### 4.4. Identification and Enrichment Analysis of DEGs

Gene expression levels across six samples were first normalized using the FPKM method [69]. DEGs between CK and PI groups were identified using the DESeq2-edgeR algorithm with thresholds of |log_2_(fold change)| ≥ 2 and *p-*value < 0.05. Gene ontology (GO) and KEGG enrichment analysis of DEGs were performed using the BMKCloud platform (https://www.bioinformatics.com.cn/) (*q* < 0.05). All transcripts were also functionally annotated using Mercator v4.0, and DEGs were also subjected to PageMan enrichment analysis using MapMan v3.6.0.

### 4.5. Gene Expression Validation by Using Quantitative Real-Time PCR (qRT-PCR)

Fifteen DEGs involved in significantly enriched pathways were selected and subjected to qRT-PCR verification. Gene-specific primers were designed using Primer3 v0.4.0 under default parameters (Supplementary Table S12). The qRT-PCR analysis was conducted according to Zhang et al. [70], with *gapdh* as the internal reference gene [71]. Relative expression levels of selected genes in CK and PI roots were calculated using the 2^−ΔΔCT^ method, with three biological replicates.

### 4.6. Determination of ACP Activity and Mineral Element Contents in Blueberry Roots

The root ACP activity was determined using a detection kit produced by Beijing Solarbio Science & Technology Co., Ltd. (Beijing, China). Nitrogen content in blueberry roots was analyzed through sulfuric acid digestion coupled with the Kjeldahl method [72]. Phosphorus and potassium contents in blueberry roots were determined using sodium bicarbonate extraction followed by the molybdenum–antimony anti-spectrophotometric method [73], and ammonium acetate extraction coupled with flame photometry [74], respectively. Calcium and magnesium contents in blueberry roots were determined using HNO_3_-HClO_4_ digestion coupled with flame atomic absorption spectrophotometry [75]. All these parameters were measured with three biological replicates.

### 4.7. Measurement of Sucrose, Starch, Total Phenolic, Total Flavonoids, and Phytohormones Contents in Blueberry Roots

Root sucrose and starch contents in blueberry roots were quantified using commercial kits produced by Solarbio Science & Technology Co., Ltd. (Beijing, China). The total phenolic and flavonoid contents in blueberry roots were determined using the Folin–Ciocalteu method [76] and the aluminum chloride colorimetric method [77], respectively. Endogenous contents of indole-3-acetic acid (IAA), jasmonic acid (JA), abscisic acid (ABA), 1-aminocyclopropane-1-carboxylic acid (ACC), and strigolactone (SL) in roots were measured using plant-specific enzyme-linked immunosorbent assay kits (Tongwei Biotechnology Co., Ltd., Shanghai, China). All these parameters were analyzed with four biological replicates.

### 4.8. Root Carotenoids Profiling and Analysis

Carotenoids in roots of CK and PI groups were further analyzed using an AB Sciex QTRAP 6500 LC-MS/MS (Framingham, MA, USA) system. For carotenoids profiling, a YMC C30 column (3 μm, 2.0 mm × 100 mm) was employed. The mobile phase comprised methanol/acetonitrile (1:3, *v*/*v*) with 0.01% BHT and 0.1% formic acid (A), and methyl tert-butyl ether with 0.01% BHT (B). The gradient program initiated at 100% A/0% B (*v*/*v*) for 3 min, linearly increased to 30% A/70% B (*v*/*v*) at 5 min, further raised to 5% A/95% B (*v*/*v*) at 9 min, held for 1 min for complete elution, then rapidly reverted to the initial conditions (100% A/0% B) and equilibrated until 11 min. The flow rate was set to 0.8 mL/min with a 2 μL injection volume.

### 4.9. Statistical Analysis

All data were organized using Microsoft Excel 2021 and are presented as mean ± SD from at least three biological replicates. SPSS 22.0 (IBM corporation, Armonk, NY, USA) was used to analyze the significance of differences of the measured parameters between CK and PI at the *p* < 0.05 level (Student’s *t-*test). For figure drawing, GraphPad Prism 8 was used.

## 5. Conclusions

**Figure 6:**
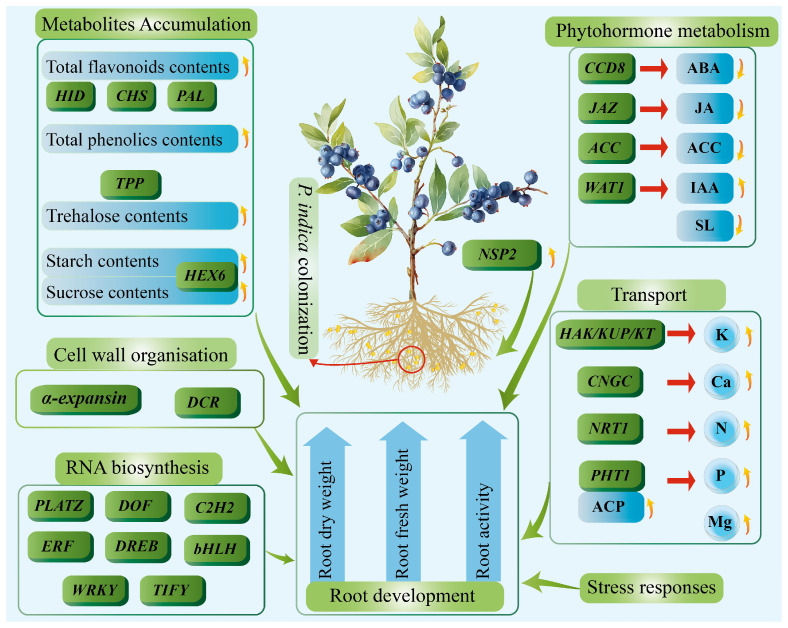
Influences of *P. indica* on the root development of blueberries under long-term tap water irrigation. *P. indica* greatly influences primary and secondary metabolites accumulation, cell wall organization, and RNA biosynthesis, and alters the phytohormone metabolism, nutrient transportation, and stress responses, thereby promoting blueberry root development. DEGs are shown in green boxes, and significantly changed biochemical parameters are shown in blue boxes/circles/arrows. Green arrows represent promoting effects on their pointed biological processes. Upward and downward yellow arrows indicate up-regulation and down-regulation, respectively.

Our study demonstrated that *P. indica* can promote blueberry root development under long-term tap water irrigation, which can cause mild salt stress in blueberry plants. Integrated transcriptomic and biochemical analyses revealed that fungal colonization enhanced blueberry root adaptability to tap water-induced mild salt stress by modulating cell wall metabolism, phytohormone metabolism, metabolites accumulation, stress responses, and nutrient transportation networks (Figure 6). Our study provides a theoretical foundation for the application of *P. indica* in blueberry cultivation.

## Figures and Tables

**Table 1:** Effects of *P. indica* colonization on the root development of blueberry plants under long-term tap water irrigation. CK: non-colonized controls; PI: *P. indica*-colonized blueberry plants. Different lowercase letters in the same line represent significant difference between CK and PI groups.

Root Growth Parameters	CK Group	PI Group
Root fresh weight (g)	72.33 ± 7.76 b	100.66 ± 5.85 a
Root dry weight (g)	26.33 ± 3.05 a	32.66 ± 4.04 a
Root activity (μg·g^−1^·h^−1^)	4.15 ± 0.55 a	5.85 ± 1.01 a

**Table 2:** Significantly enriched BIN pathways by the DEGs identified between CK and PI.

BIN ID	BIN Name	All DEGs	Up	Down	*p*-Value
15.5.7	RNA biosynthesis; transcriptional regulation; and AP2/ERF transcription factor superfamily.	73	6	67	1.19 × 10^−4^
18.4.1.12	Protein modification; phosphorylation.; TKL protein kinase superfamily; and protein kinase (LRR-XII).	18	15	3	1.19 × 10^−4^
24.1.2	Solute transport; primary active transport; and P-type ATPase superfamily.	20	0	20	1.19 × 10^−4^
24.1.2.2	Solute transport; primary active transport; P-type ATPase superfamily; and P2 family.	20	0	20	1.19 × 10^−4^
24.1.2.2.2	Solute transport; primary active transport; P-type ATPase superfamily; P2 family; and P2B-type calcium cation-transporting ATPase (ACA).	20	0	20	1.19 × 10^−4^
15.5.1.5	RNA biosynthesis; transcriptional regulation; C2C2 transcription factor superfamily; and transcription factor (DOF).	9	9	0	8.34 × 10^−4^
21.4.2.1	Cell wall organization; cell wall proteins; expansin activities; and alpha-class expansin	10	10	0	8.48 × 10^−4^
15.5.7.2	RNA biosynthesis; transcriptional regulation; AP2/ERF transcription factor superfamily; and transcription factor (DREB).	43	3	40	1.98 × 10^−3^
11.9	Phytohormone action and strigolactone.	8	8	0	2.50 × 10^−3^
24.1	Solute transport and primary active transport.	44	1	43	1.01 × 10^−3^
24.2.1.1	Solute transport; carrier-mediated transport; DMT superfamily; and NST-TPT group.	13	0	13	1.01 × 10^−2^
24.2.1.5	Solute transport; carrier-mediated transport; DMT superfamily; and solute transporter (UmamiT).	7	7	0	1.01 × 10^−2^
11.7.3.3	Phytohormone action; jasmonic acid; conjugation and degradation; and jasmonoyl-amino acid carboxylase (CYP94C).	5	0	5	1.01 × 10^−2^
15.5.42	RNA biosynthesis; transcriptional regulation; and transcription factor (TIFY).	15	0	15	1.01 × 10^−2^
7.9.6	Coenzyme metabolism; NAD/NADP biosynthesis; and NAD homeostasis.	7	0	7	1.19 × 10^−2^
7.9.6.2	Coenzyme metabolism; NAD/NADP biosynthesis; NAD homeostasis; and pyrophosphohydrolase (NUDX).	7	0	7	1.19 × 10^−2^
3.3	Carbohydrate metabolism and trehalose metabolism.	7	0	7	1.49 × 10^−2^
3.3.2	Carbohydrate metabolism; trehalose metabolism; and trehalose-6-phosphate phosphatase.	7	0	7	1.49 × 10^−2^
15.5.40	RNA biosynthesis; transcriptional regulation; and transcription factor (PLATZ).	6	6	0	1.66 × 10^−2^
15.5.1	RNA biosynthesis; transcriptional regulation; and C2C2 transcription factor superfamily.	16	10	6	1.92 × 10^−2^
27.1.4	Multi-process regulation; circadian clock system; and time-of-day-dependent expressed repressor (PRR).	7	7	0	2.04 × 10^−2^
24.2.1.1.11	Solute transport; carrier-mediated transport; DMT superfamily; NST-TPT group; and nucleotide sugar transporter (UUAT).	9	0	9	2.46 × 10^−2^
21.4.2	Cell wall organization; cell wall proteins; and expansin activities.	19	10	9	2.46 × 10^−2^
19.2.2.1.4.2	Protein homeostasis; ubiquitin–proteasome system; and ubiquitin-fold protein conjugation; ubiquitin conjugation (ubiquitylation); ubiquitin-ligase E3 activities; and U-Box E3 ligase activities.	49	0	49	3.28 × 10^−2^
26.10.1.6	External stimuli response; symbiont; common symbiotic signaling pathway (CSSP); and NSP1-NSP2 nodulation initiation complex.	4	4	0	3.28 × 10^−2^
26.10.1.6.2	External stimuli response; symbiont; common symbiotic signaling pathway (CSSP); NSP1-NSP2 nodulation initiation complex; and component NSP2.	4	4	0	3.28 × 10^−2^
27.1	Multi-process regulation and circadian clock system.	9	7	2	3.51 × 10^−2^
26.4	External stimuli response and temperature.	17	0	17	3.81 × 10^−2^
26.4.3	External stimuli response; temperature; and cold response.	17	0	17	3.81 × 10^−2^
15.5.22	RNA biosynthesis; transcriptional regulation; and WRKY transcription factor activity.	41	0	41	3.82 × 10^−2^
15.5.22.1	RNA biosynthesis; transcriptional regulation; WRKY transcription factor activity; and transcription factor (WRKY).	41	0	41	3.82 × 10^−2^
9.1.4	Secondary metabolism; terpenoids; and terpene biosynthesis.	14	0	14	3.92 × 10^−2^
26.4.3.4	External stimuli response; temperature; cold response; and ICE-CBF cold acclimation transcriptional cascade.	14	0	14	4.09 × 10^−2^
26.4.3.4.2	External stimuli response; temperature; cold response; ICE-CBF cold acclimation transcriptional cascade; and transcription factor (CBF/DREB1).	14	0	14	4.09 × 10^−2^
15.5.30	RNA biosynthesis; transcriptional regulation; and transcription factor (bHLH).	21	1	20	4.82 × 10^−2^

## Data Availability

The original contributions presented in the study are included in the article/supplementary material, further inquiries can be directed to the corresponding author.

## Supplementary Materials

The following supporting information can be downloaded at: https://www.mdpi.com/article/10.3390/plants14233646/s1, Figure S1: PCR detection results of *P. indica* colonization in blueberry roots. M: DL2000 DNA marker; P: positive control for the 250 bp *Pitef1* amplicon; 1-3: non-inoculated control; 4-6: genomic DNA from *P. indica-*colonized blueberry roots; Table S1: The quality indicators of the transcriptome sequencing data used in this study; Table S2: GO enrichment analysis of the DEGs between CK and PI roots; Table S3: MapMan functional annotation and classification results of DEGs; Table S4: DEGs enriched in the RNA biosynthesis pathway; Table S5: DEGs enriched in the solute transport pathway; Table S6: DEGs enriched in the external stimuli response pathway; Table S7: DEGs enriched in the phytohormone action pathway; Table S8: DEGs enriched in the carbohydrate metabolism; Table S9: DEGs enriched in the cell wall organization pathway; Table S10: DEGs enriched in the secondary metabolite biosynthesis pathway; Table S11: Carotenoids in roots of *P. indica-*colonized and non-colonized blueberries; Table S12: Information of the primers used for the expression validation of the 15 selected DEGs.

## Abbreviations

The following abbreviations are used in this manuscript:

ABA	Abscisic acid
ACC	1-aminocyclopropane-1-carboxylic acid
ACP	Acid phosphatase
AMF	Arbuscular mycorrhizal fungus
bHLH	Basic helix–loop–helix
BP	Biological processes
bp	Base pair
Ca	Calcium
CC	Cellular components
CCD8	Carotenoid cleavage dioxygenase 8
cDNA	Complementary DNA
C2H2	Cys2-His2 zinc finger
CHS	Chalcone synthase
CNGC	Cyclic nucleotide-gated channel
DEGs	Differentially expressed genes
DOF	DNA-binding with one finger
DREB	Dehydration-responsive element binding protein
EC	Electrical conductivity
ERF	Ethylene response factor
EXPA	Alpha-class expansin
Gb	Gigabase
GO	Gene ontology
HEX6	Hexose carrier 6
HID	2-hydroxyisoflavone dehydratase
IAA	Indole-3-acetic acid
JA	Jasmonic acid
K	Potassium
KEGG	Kyoto Encyclopedia of Genes and Genomes
LRR-XII	Leucine-rich repeat receptor kinases
MF	Molecular function
Mg	Magnesium
N	Nitrogen
NSP2	Nodulation signaling pathway 2
P	Phosphorus
PAL	Phenylalanine ammonia-lyase
PI	*Piriformospora indica*-colonized
PLATZ	Plant AT-rich sequence and zinc-binding protein
qRT-PCR	Quantitative real-time PCR
SL	Strigolactone
TF	Transcription factor
TPP	Trehalose-phosphate phosphatase
TTC	Triphenyltetrazolium chloride
WAT1	Wall are thin 1
