# Supplementary material for: Integrated Transcriptomic and Biochemical Analyses Reveal the Root Development-Promoting Mechanism of Piriformospora indica on Blueberry Under Tap Water Irrigation
Source: Plants (Basel). 2025 Nov 29;14(23):3646. doi: 10.3390/plants14233646 (PMC12694296; doi:10.3390/plants14233646)
Supplement: Supplementary file 1 [file plants-14-03646-s001.zip › Figure S1.pdf]

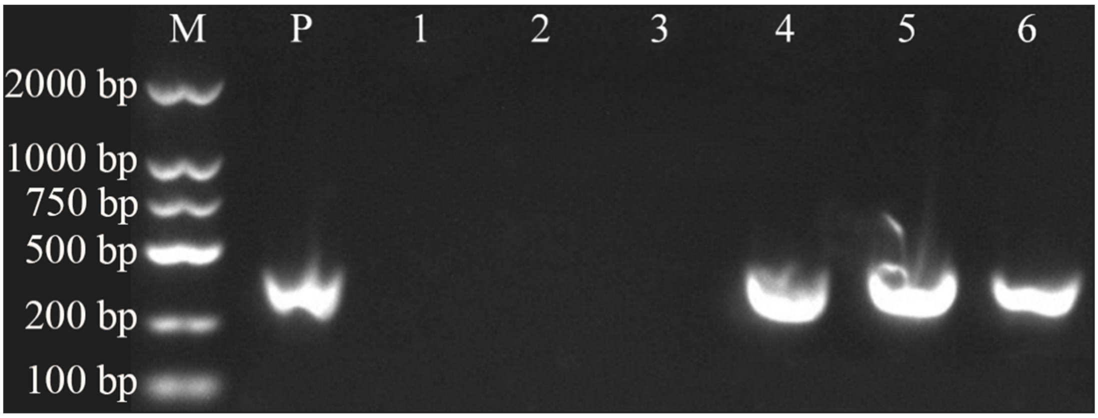

Figure S1. PCR detection results of *P. indica* colonization in blueberry roots. M: DL2000 DNA marker; P: positive control for the 250 bp *Pitef1* amplicon; 1-3: non-inoculated control; 4-6: genomic DNA from *P. indica*-colonized blueberry roots.
